# Spaceflight Induced Disorders: Potential Nutritional Countermeasures

**DOI:** 10.3389/fbioe.2021.666683

**Published:** 2021-04-21

**Authors:** Fabio Costa, Francesco Saverio Ambesi-Impiombato, Tommaso Beccari, Carmela Conte, Samuela Cataldi, Francesco Curcio, Elisabetta Albi

**Affiliations:** ^1^Department of Pharmaceutical Sciences, University of Perugia, Perugia, Italy; ^2^Dipartimento di Area Medica, University of Udine, Udine, Italy

**Keywords:** microgravity, energy intake, life-style, nutrition, spaceflight

## Abstract

Space travel is an extreme experience even for the astronaut who has received extensive basic training in various fields, from aeronautics to engineering, from medicine to physics and biology. Microgravity puts a strain on members of space crews, both physically and mentally: short-term or long-term travel in orbit the International Space Station may have serious repercussions on the human body, which may undergo physiological changes affecting almost all organs and systems, particularly at the muscular, cardiovascular and bone compartments. This review aims to highlight recent studies describing damages of human body induced by the space environment for microgravity, and radiation. All novel conditions, to ally unknown to the Darwinian selection strategies on Earth, to which we should add the psychological stress that astronauts suffer due to the inevitable forced cohabitation in claustrophobic environments, the deprivation from their affections and the need to adapt to a new lifestyle with molecular changes due to the confinement. In this context, significant nutritional deficiencies with consequent molecular mechanism changes in the cells that induce to the onset of physiological and cognitive impairment have been considered.

## Introduction

The space environment induces cellular and molecular changes with consequents damages in different organs and tissues of astronauts. Due to the complexity of the space environment it is really difficult to distinguish damages induced by gravity, those induced by radiation and those caused by confinement. Therefore, simulated microgravity experiments are really useful for discriminating the causes of damage. It is worth considering that numerous studies conducted in space have focused attention on the absence of gravity. The effects of microgravity on humans, animals, plants, and objects in spacecrafts are evident at a macroscopic level, when they float freely within the cabin. But a vast variety of effects invisible to the naked eye have been described at the cellular and molecular levels that induce disorders in different astronaut systems. During the first few hours of flight, it is quite common for the astronauts to experience multiple symptoms of the so-called *Space Adaptation Syndrome*, especially in the course of their first mission ever. The most common of those symptoms include nausea and vomiting, diarrhea, dizziness, headache, lethargy and general malaise, frequently culminating with the manifestation of a marked lack of appetite which may last for several days from the beginning of the mission (Kornilova and Kozlovskaya, 2003). This, combined with other factors such as the deleterious effect of radiation on the body, the less-than-optimal quality of food and the altered perception of smells and flavors, contribute to establishing an inadequate energy intake, which may then lead to the manifestation of the most significant side effects of long-term weightlessness, such as muscle atrophy and deterioration of the skeleton, and thyroid dysfunction (Albi et al., 2017). Furthermore, the redistribution of body fluids to the upper portion of the body which causes the “moon face” aspect, a slowdown in the functionality of the cardiovascular system and blood flow, a reduction of red blood cells, neuro-immuno-endocrine-metabolic interaction disorders (Strollo et al., 2018). Although most of these effects may quickly recede upon return to Earth, they may nevertheless affect the health and performance of the crewmembers due to the changes of molecular mechanisms in the cells. Operational improvements and biomedical countermeasures have been used over time to limit molecular changes and consequently improve the health of astronauts (Crucian et al., 2020). However, many molecular mechanisms induced by the space environment are still unexplored and research in the field is rapidly expanding.

### Impact of Space Environment on Health

The space environment is responsible for damages in different organs and tissues due to microgravity and radiation (Stein and Leskiw, 2000). The main pathogenetic mechanism is the oxidative stress. It is known that on Earth, body cells are constantly subjected to the action of Reactive Oxygen Species (ROS) which are produced as a result of both cellular metabolism and exposure to external agents such as X rays, UV, pollution, cigarette smoke, etc. Usually, ROS production is balanced by the activation of specific biochemical detoxification systems. If high levels of ROS are produced, cell damage is proportionally induced. Exposure to space environment is associated with increased oxidative stress on membrane lipids, proteins, and specifically on DNA which might cause permanent damage in the genetic code of tissues and cells, leading to cell death and cancer (Tominaga et al., 2004). Yatagai et al. (2019) proposed that interaction between microgravity and space radiation targets many molecular mechanisms in addiction to ROS signal as DNA repair, replication, transcription, gene and protein expression.

#### Humans

*Astronauts* who participated in long-term missions aboard the ISS were exposed to multiple stress factors with repercussions on cardiovascular health (Table 1). In fact, changes in gravitational forces, alteration of physical activity patterns and metabolic stress factors were associated with increased blood concentrations of markers indicative of vascular growth, inflammation and oxidative stress, which have a negative impact on the structural and functional aspects of the vascular system, such as accelerated stiffening of the arterial walls and subsequent development of arteriosclerosis in association with insulin resistance (Hughson et al., 2016). A relevant study on the effect of microgravity in cardiovascular system during long stay in space was performed in two homozygous American astronauts, Mark and Scott Kelly (Garrett-Bakelman et al., 2019). While Mark remained on Earth, Scott spent nearly a year (342 days) aboard the ISS. The study compared physiological, genetic and behavioral parameters of two twins, aiming to evaluate the changes of the cardiovascular system and the probability of arteriosclerosis onset, by ultrasound examination of their arteries. During the mission, and immediately after Scott’s return to Earth, a thickening of the carotid artery was observed, also confirmed by the increase in biological markers of inflammation, such as the inflammatory cytokine *IL-1ra*. Fortunately, most of these cardiovascular changes returned to normal within approximately 6 months. In addition, doctor James Thomas has shown that after 6 months in space the astronaut’s heart typically become more spheroidal in shape, possibly because it worked less harshly in microgravity conditions (May et al., 2014). The team of NASA researchers led by Thomas taught astronauts to take images of their heart, using ultrasonic equipment installed on the ISS, before, during and after the mission. Data showed that in the absence of gravity the astronaut’s heart became more spherical by 9.4%, a transformation similar to what researchers had predicted in the past, using mathematical models. However, this phenomenon seemed to be temporary as, upon returning to Earth, the hear quickly regained its original elongated shape (May et al., 2014).

**TABLE 1:**
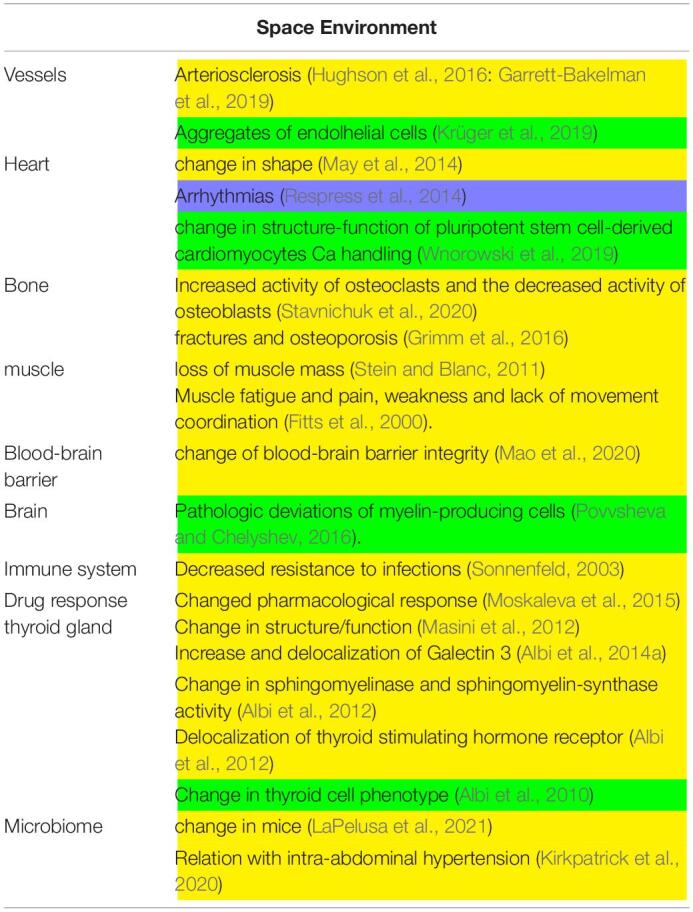
Main experiments conducted in space environment. In yellow, *in vivo* experiments; in green, *in vitro* experiments.

The space environment also affects bone structure and function. Bones play different roles, from supporting body weight to mineral reserves, acid-base homeostasis and bone marrow containment and protection. These functions are all affected by microgravity (Stavnichuk et al., 2020). In physiological conditions on Earth, the osteoclast activity with consequent bone resorption and the osteoblast activity with consequent bone tissue production are balanced. Bone loss occurs during spaceflight, due to the combined effect of the increased activity of osteoclasts and the decreased activity of osteoblasts, resulting in negative calcium balance and consequent bone loss (Stavnichuk et al., 2020). Calcium is thus released into the bloodstream and urine, increasing the risk of kidney stone formation (Stavnichuk et al., 2020). The calcium release from the bone suppresses the parathyroid hormone (PTH) with consequent reduction of vitamin D3 activation in the kidney. Low level of activated vitamin D3 (1,25-dihydroxyvitamin D3) is in turn responsible for the reduction in calcium absorption at the gastrointestinal level. Notably, short and long term space missions might significantly increase astronaut’s health risks in term of fractures or osteoporosis (Grimm et al., 2016).

Moreover, spaceflight missions induce the loss of body mass. Most muscle loss, representing a relevant contribution in such loss, generally occurs in the early mission stages, during the critical adaptation period of the human body to microgravity. After spending several months in space, the loss of muscle mass can be significant, in the order of 2.4%/100 mission days (Stein and Blanc, 2011). As in microgravity conditions movements generally require minimal effort, the limited use of force causes the muscle to lose mass, volume and efficiency, particularly in the lower limbs (Stein and Blanc, 2011). Such changes may represent a problem upon returning to Earth as the structurally modified muscles require more energy, to the point that even maintaining the upright position may be difficult. The recovery time on Earth is proportional to the length of the flight, so astronauts inevitably have to undergo very rigorous physical training sessions before, during and after the flight (Fitts et al., 2000). Astronauts returning from even short space flights, may experience muscle fatigue and pain, weakness and lack of movement coordination (Fitts et al., 2000).

#### Animals

Mice involved in experiments conducted during space mission suffered from a stressful condition that was responsible for the alteration of the blood-brain barrier integrity (Mao et al., 2020; Table 1). Morover, Sonnenfeld (2003) reported that spaceflight results in decreased resistance to infection in animals. Interestingly, significant changes in the members of cytochrome P450 superfamily such as CYP2C29, CYP2E1, and CYP1A2, involved in drug metabolism, were detected in mice after spaceflights indicating a modified pharmacological response (Moskaleva et al., 2015). In a truly unique experiment of longest stay of mice on board of the International Space Station (ISS, 91 days) inside the “Mouse Drawer System, a facility built by “Thales Alenia Space” for the “Agenzia Spaziale Italiana,” Masini et al. (2012) demonstrated a change of structure/function of thyroid gland (Masini et al., 2012). The thyroid gland presented also changes in the expression and localization of Galectin-3, indicating the thyroid cell transformation (Albi et al., 2014b). Moreover, in the thyroid tissue it was evident an overexpression of enzymes for sphingomyelin metabolism such as sphingomyelinase and sphingomyelin-synthase and, of thyroid stimulating hormone receptor (Albi et al., 2012). It is relevant that these results were opposite to those obtained in hypergravity condition (Albi et al., 2014a) and different from changes induced by UV radiation exposure (Albi et al., 2009). Interestingly, a change in an elevated microbiome alpha diversity and an altered microbial community structure were reported in mice after a 37-day spaceflight onboard the ISS (LaPelusa et al., 2021). In a recent review by Kirkpatrick et al. (2020), the evidence from animal models indicating that intra-abdominal hypertension affects the intestinal microbiota in space missions is reported.

#### Cells

Studies of cell cultures in space require specific devices considering cell adaptation and reaction to space environment (Table 1). For example, endothelial cells form aggregate due to enhanced collagen and laminin (Krüger et al., 2019), pluripotent stem cell-derived cardiomyocytes Ca handling change in structure-function (Wnorowski et al., 2019) and, myelin-producing cells induce pathologic deviations in space flight (Povvsheva and Chelyshev, 2016). Morevover, it has been demonstrated that proliferating thyroid cells behave as quiescent cells in the International Space Station after undergoing change in sphingolipid metabolism (Albi et al., 2010).

### Changes of Molecular Mechanisms Induced by Simulated Microgravity

The effects of microgravity on intracellular molecular mechanisms are more easily studied with simulated microgravity than with real microgravity during spaceflights in both animals and cells (Table 2).

**TABLE 2:**
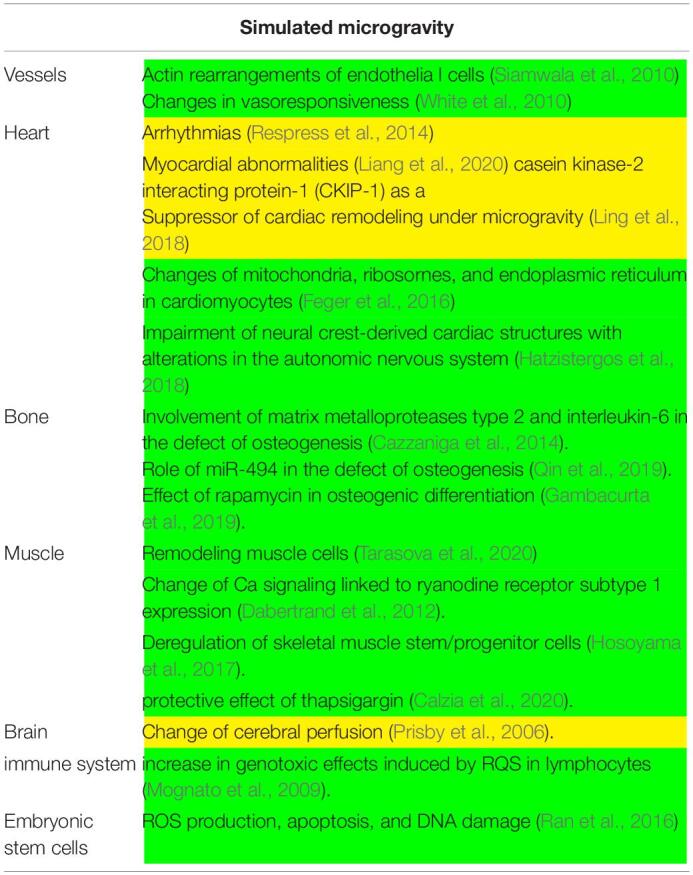
Main experiments conducted in simulated microgravity. In yellow, *in vivo* experiments; in green, *in vitro* experiments.

#### Animals

In simulated microgravity, alterations in the nitric oxide synthase signaling mechanism is responsible for vasodilation of cerebral arteries with increase in cerebral vascular resistance and reductions in cerebral perfusion in mice (Prisby et al., 2006). Several lines of evidence suggested that, in addition to vascular remodeling, microgravity induces cardiac remodeling, including atrophy and dysfunction, that is related to: (1) abnormal intracellular Ca regulation consequent to the ryanodine receptor phosphorylation (Respress et al., 2014) that is responsible for arrythmias; (2) action of calpain on p47 *^*phox*^* phosphorylation via ERK1/2 and p38 pathways that induces myocardial abnormalities (Liang et al., 2020). Interestingly, Ling et al. (2018) reported that casein kinase-2 interacting protein-1 (CKIP-1) is a suppressor of cardiac remodeling under microgravity.

#### Cells

Simulated microgravity can potentiate the effects of H_2_O_2_ on ROS production, apoptosis, and DNA damage in mouse embryonic stem cells (Ran et al., 2016). Moreover, it delays the rejoining of double-strand breaks and increase the genotoxic effects induced by ROS after radiation treatment in human lymphocytes (Mognato et al., 2009). Also changes of protein content and function of the mitochondria, ribosomes, and endoplasmic reticulum of rat neonatal cardiomyocytes have been reported (Feger et al., 2016). Hatzistergos et al., 2018 described as impairment of neural crest-derived cardiac cells with consequent alterations in neurogenesis.

Siamwala et al. (2010) demonstrated that microgravity modulates endothelial actin rearrangements with releasing nitric oxide by causing vascular endothelium remodeling. In support, White et al. (2010) reported that changes in vasoresponsiveness under microgravity is due to endothelial-dependent nitric oxide/cGMP pathway.

Interestingly, in microgravity, rapamycin induces transcriptional activation of blood-derived stem cells towards osteogenic differentiation by influencing bone formation (Gambacurta et al., 2019). Moreover, due to the delay of microvascular endothelial cell growth and the release of a high amounts of matrix metalloproteases type 2 and interleukin-6, the growth of osteoblasts is retarded and their osteogenic activity is impaired (Cazzaniga et al., 2014). Also miR-494 is correlated with a decrease in osteogenesis due to a marked reduction in osteoblast differentiation genes (Qin et al., 2019).

The muscular symptomatology is linked to a remodeling of the skeletal muscle (Tarasova et al., 2020) linked to an adaptation of Ca signaling pathways by the regulation of the ryanodine receptor subtype 1 expression (Dabertrand et al., 2012). This perturbation can be preventing by treating cells with thapsigargin that hinders the segregation of calcium ions in the mitochondria and in the sarco/endoplasmic reticula (Calzia et al., 2020). Moreover, microgravity was found to deregulate skeletal muscle stem/progenitor cells pool due to inhibition of the TRAF6/ERK pathway with consequent Pax7 down-expression (Hosoyama et al., 2017).

## Effect of Microgravity on Energy Balance

### Space Environment

**FIGURE 1:**
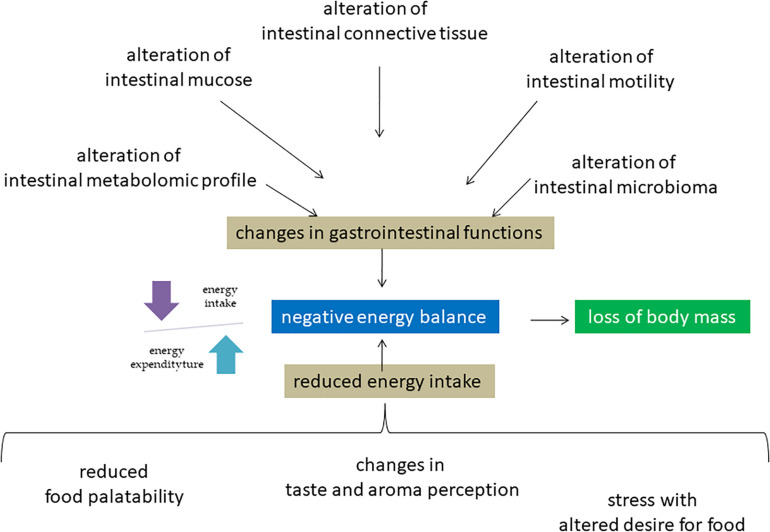
Molecular mechanisms of negative energy balance of astronauts in space environment. Microgravity induces a redistribution of body fluids with consequences on different molecular mechanisms in including intestinal system, sense organs and nerve center of hunger. The consequent negative energy balance is responsible for the disorders of different systems including muscle and bone leading to loss of body mass.

There is accumulating evidence supporting the relevance of balancing total energy intake and energy expenditure during space missions (Figure 1). If the food energy intake is not adequate to the physiological demands, the high energy expenditure may lead to a negative energy balance (Stein, 2013). Adequate energy intake is certainly the most important aspect of astronaut’s nutrition. During spaceflight history it was not always possible to reach optimal quantitative standards relating to the physiological energy demands of crewmembers. Lower energy inputs compared to the requirements may be attributed not only to the food availability and palatability, but also to the individual changes in taste and aroma perception, which often occur during flight missions. This is possibly caused by the body fluids redistribution with a significant shift towards the upper part of the body, causing swelling of the face and paranasal sinuses.

This may induce molecular changes in sensory cells with alteration of taste and smell resulting in reduced food cravings. However, the astronauts’ energy intake abroad the ISS has been progressively increased over the years, in line with better food palatability achieved through the improvement of food preparation and preservation techniques and through significant reformulations of space food products. These factors, together with our improved notions on food and nutrition, developed in parallel with the evolution of space exploration and thanks to the contribution from numerous crew members, lead us to the present situation. It is a fact that many astronauts of the ISS daily assume their correct energy supply, thus managing to counterbalance, for the most part at least, their loss of body mass (Smith et al., 2005). Most recently, molecular mechanisms have been clarified. Changes in gastrointestinal functions have been also observed during flight due to the redistribution of body fluids in combination with a reduced fluids and dietary intake suffering. Consequently, a decrease in the gastrointestinal motility with manifest altered secretion of gastric acid, of the rhythmic contractions of the stomach and intestines with impairment of gastric emptying.

In addition to all of the above, frequently astronauts are affected by motion sickness. All this negatively interferes with the health and well-being of the astronauts, mainly during the first days of the mission, as motion sickness typically disappear after the first few days, albeit the reduced dietary intake may extend even beyond the first week (Smith et al., 2005). Often astronauts have a dietary preference for carbohydrates rather than fats. This phenomenon seems to be due to a physiological response to stress characterized by an increase in the brain levels of tryptophan, a precursor of serotonin which may be classified as an anorexic agent (Da Silva et al., 2002). Clinical studies and laboratory tests clearly indicate that food intake and body weight loss in microgravity conditions are regulated by a complex mechanism of neuroendocrine changes, such as the levels of leptin, an anorexigenic hormone secreted by the adipose tissue that increases satiety and reduces food intake (Smith et al., 2001). Bergouignan et al. (2016) reported an increase in plasma concentration of Glucagon-like peptide-1 (GLP-1), a satiety hormone produced by the intestine which slows gastric emptying by increasing the sense of satiety and reducing the appetite by acting directly on the central nervous system. In addition, no variations in the plasma concentration of ghrelin, an orexigenic hormone produced by the stomach, were found (Lane et al., 2013). In overall, these fluctuations in plasma hormone concentrations influence molecular mechanisms of hunger regulation in the nervous system cells that contribute to the reduction of appetite. Consequences related to a long-term low calorie intake lead to an impairment of the functionality of body systems above reported. In addiction, a specific consequence is the impairment of the cognitive functions, which could significantly compromise the astronaut’s ability to carry out their work and performance in orbit (Mammarella, 2020). Malnutrition in the space environment is still under investigation and discussion. In numerous studies, conflicting results have already been reported (Lang et al., 2017). Smith et al. (2005) found an inadequate intake of minerals (calcium, potassium, and sodium) and of oligo-elements (iron) together with deficiencies in vitamins K and D. Then, Zwart et al. (2011) indicated that the vitamin K turnover is unchanged in space. Considering the defect of microbioma in space environment above reported, its involvement in nutrient deficiency might be relevant in space biomedicine.

### Simulated Microgravity

Most recently, molecular mechanisms have been clarified. In microgravity condition, the intestinal mucosal cells are destroyed due to up-regulation of pro-apoptotic protein Bax and down-regulation of anti-apoptotic protein Bcl2 (Jin et al., 2018). Intestinal barrier dysfunction is also mediated by myosin light chain kinase (Wang S. et al., 2020). Atiakshin et al. (2019) reported a change of fibrous structure of extracellular matrix of the connective tissue of the digestive system organs. Moreover, weightlessness influences the intestinal metabolomic profile and, a strong relation between dysbiosis and altered glucose metabolism-related genes in the hind limb-unloading mouse model was described (Jin et al., 2019; Wang et al., 2019).

### Nutrients to Counteract Spaceflight Damages

Obviously, specific nutrients are useful to counteract damages induced by microgravity as discussed above (oxidative stress, cardiovascular system, bone, muscle, etc.). To at least contain the increase in oxidative stress severity during space missions, it would be important to increase the dietary intake of many food antioxidants, in particular vitamins and minerals, such as vitamin E, vitamin A, vitamin C, omega-3 fatty acids, copper, zinc, manganese, selenium and iron (Sacheck and Blumberg, 2001). The combination of dietary defenses and endogenous antioxidants production, seems to play a significant protective role against oxidative damages (Gao and Chilibeck, 2020). Protection from cardiovascular problems may be obtained with low-glycemic index diets because bed rest reportedly induces glucose intolerance (Wang X. P. et al., 2020). As regards to bone damages, particular attention was paid to vitamin D3. As this particular vitamin cannot be endogenously synthesized due to lack of UV exposure, the decrease in vitamin D3 serum concentration is a serious concern for space exploration missions, particularly long-term. However, it has been reported that dietary intake of vitamin D3 in the form of supplements is not the answer to microgravity-mediated bone density loss since, if administered in large doses, vitamin D3 may induce hypercalcemia, kidney stones, and irreversible calcification of soft tissues (Vieth, 1999). It has been extensively demonstrated that microgravity impacts the proteome in humans with consequent changes in various biological processes such as angiogenesis, apoptosis, cell adhesion, migration, proliferation, stress response, and signal transduction (Strauch et al., 2019). In thyroid cells, microgravity changes the expression and localization of the specific protein Galectin-3 (Albi et al., 2014b) and also induces the overexpression of enzymes such as sphingomyelinase and sphingomyelin-synthase and of the thyroid stimulating hormone receptor (Albi et al., 2012), the opposite to what happens in hypergravity (Albi et al., 2014a) and different from changes induced by radiation exposure (Albi et al., 2009). In overall, significant protein remodeling is induced specifically by microgravity.

## Discussion

Exposure to space environment is not without risk, both during and after the actual exposure. Since the first missions accomplished the last century many and considerable advances have been made both in the medical and nutritional fields, as well as in technological research. Thus, the study of the relationship between damage induced by the spatial environment in the cardio-circulatory, bone and muscular system together to the stress condition due to the confinement and the alteration of the energy balance due to alterations of the gastrointestinal system, alteration of the sense organs and nutritional deficits represents an interesting field of investigation for the health of astronauts. For over 50 years scientist cooks, nutritionists and experts in various fields have significantly advanced their knowledge and insights, gradually improving the quality and nutritional values of Space Food, preparing progressively better-tasting and nutritious meals for astronauts through increasingly advanced technologies, challenging the difficulties and pitfalls that the extreme space environment. Nowadays the availability, characteristics and organoleptic properties, storage and preparation techniques of space food have reached significant high quality standards. Today on the ISS, the United States provides about half of the food while other countries, including Italy, provide the rest. Since it is not possible to use open flames on the ISS, foods are dehydrated, freeze-dried, pre-cooked or autoclaved and then stored in aluminum bags or cans at room temperature, to minimize the alteration of their nutritional properties, appearance and taste. In this way, space food has a shelf-life of approximately 18–24 months. Astronauts simply add hot or cold water, thus allowing the chosen serving to (hopefully) regain its original aspect and nutritional properties. Current studies and technologies aim to make the daily life of space crews as close as possible to life on Earth, trying to keep the habits related to personal care unchanged also through the art of food supply (“from the earth to the plate”). For astronauts, in fact, it is of great importance to be able to have constant supplies of foods as close as possible to the natural version and then to consume them together with the other crewmembers, also because such activity may provide significant psychological benefits. In the review, we explored the emerging insights into the system disorders in space environment highlighting nutritional deficiencies that might activate molecular mechanisms of damage.

## Conclusion

Further studies of the metabolic modifications of nutrients in the human organism in microgravity conditions will be relevant to provide diets increasingly suited to the metabolic needs of the astronauts, in order to prevent damages to their organs and tissues in the extreme space environment.

## Author Contributions

FCo and EA: conceptualization and writing. FCu, CC, TB, and FA-I: review and editing. All authors contributed to the article and approved the submitted version.

## Conflict of Interest

The authors declare that the research was conducted in the absence of any commercial or financial relationships that could be construed as a potential conflict of interest.
